# An *In Vivo* Study of Self-Regulated Study Sequencing in Introductory Psychology Courses

**DOI:** 10.1371/journal.pone.0152115

**Published:** 2016-03-22

**Authors:** Paulo F. Carvalho, David W. Braithwaite, Joshua R. de Leeuw, Benjamin A. Motz, Robert L. Goldstone

**Affiliations:** 1 Department of Psychological and Brain Sciences, Indiana University, Bloomington, IN, United States of America; 2 Department of Psychology, Carnegie Mellon University, Pittsburgh, PA, United States of America; Technion Israel Institute of Technology, ISRAEL

## Abstract

Study sequence can have a profound influence on learning. In this study we investigated how students decide to sequence their study in a naturalistic context and whether their choices result in improved learning. In the study reported here, 2061 undergraduate students enrolled in an Introductory Psychology course completed an online homework tutorial on measures of central tendency, a topic relevant to an exam that counted towards their grades. One group of students was enabled to choose their own study sequence during the tutorial (Self-Regulated group), while the other group of students studied the same materials in sequences chosen by other students (Yoked group). Students who chose their sequence of study showed a clear tendency to block their study by concept, and this tendency was positively associated with subsequent exam performance. In the Yoked group, study sequence had no effect on exam performance. These results suggest that despite findings that blocked study is maladaptive when assigned by an experimenter, it may actually be adaptive when chosen by the learner in a naturalistic context.

## Introduction

Changes in educational approaches and technologies have created new opportunities for learners to study in unsupervised situations where they must make active decisions about their own study. This introduces new interesting possibilities such as online and intelligent tutoring systems, but also new challenges. On the one hand, increased opportunity for self-regulated study might lead to improved engagement and better allocation of study time [1,2]. On the other hand, self-regulated learning might not lead to improvement because of deficiencies in learners’ knowledge about the efficacy of different study methods [3]. Are students able to take advantage of self-regulated study by organizing their study in the optimal way? In the present work we investigate how students decide to sequence their study and how these decisions affect their learning outcomes in an exam context.

### Why is study sequence important?

When organizing their own study, students make many consequential decisions [4]. One important decision learners must make is how to sequence the study materials in the most efficient way. Variations in study sequence can have a substantial effect on learning, even when the materials studied are kept constant [5,6]. One key sequencing decisions is whether to block examples of the same concept (studying all problems/examples of one concept before moving on) or to interleave examples from different concepts during study.

Numerous laboratory studies have found advantages for interleaved over blocked study [7–10]. For example, Kornell and Bjork [8] showed that interleaved presentation of paintings from different artists resulted in better learning of the artists’ styles and improved transfer to novel paintings, compared to presenting the artists’ paintings in separate blocks. There are several potential advantages to interleaved study. Compared to blocked study, interleaving different concepts allows for comparison of successive examples, increases the temporal delay between repetitions of the same concept (even if these are not verbatim repetitions of the same exact problem/example), and might allow students to test themselves during study (for a recent discussion of these factors, see [11]). Interleaved study may also constitute a “desirable difficulty” [12] in the sense of eliciting greater effort during study, and therefore improve long-term retention. Although all of these factors are potentially in play, in the current study we focus on the opportunity to compare successive examples from different concepts as mediating the effectiveness of interleaving [7,11,13–17]. Such comparisons should facilitate learning of discriminative features, that is, properties that critically distinguish between the two concepts.

However, sometimes the cognitive challenge is not in finding differences between concepts but finding commonalities among the different examples of the same concept. In these situations, blocked study, which promotes within-concept comparisons among temporally close examples and thus highlights within-concept similarities, may be more effective. Consistent with this view, several studies have found advantages for blocked study when between-concept differences are relatively obvious or within-concept similarities are subtle [14,15,18–20]. For example, Carpenter and Mueller [18] taught non-French speakers orthographic-to-phonological rules of different French words (i.e., “-eau” and the corresponding sound /o/ in the words “bateau”, “carreau” and “corbeau”, and “-er” and the sound /se/ in the words “adosser” or “attraper”). Learners who studied several different words containing the same rule in a blocked sequence learned the orthographic-to-phonological mapping better than learners who saw the same words interleaved by rule.

In most studies investigating the relative benefits of interleaved and blocked study, these sequences have been assigned to learners rather than self-generated. By contrast, in everyday educational settings the student is often in control of how to organize their study. Do students’ real-life study habits reflect the appropriate sequencing strategies? In laboratory settings, learners prefer a blocked sequence when given the choice to organize their own study. For instance, Tauber et al. [21] recently found that when studying examples of different bird species, learners overwhelmingly preferred to study all examples of one species before starting study of the other species of birds. This is a particularly interesting finding given that, when learners do not have a choice, interleaved study is more effective for learning the same type of stimuli (e.g., [10]).

These results are consistent with previous evidence showing that when asked which learning sequence (interleaved or blocked) learners believe would result in best learning, the majority choose blocked study, even in situations where interleaved study is more beneficial [8,20]. One possible reason for this belief is that blocked sequences may facilitate cognitive processing during study, resulting in a sense of fluency which would lead to over-estimation of how much learning is occurring [3]. Additionally, a preference for blocked study may reflect habitual biases [3,22] or a desire to avoid the greater cognitive effort associated with interleaving [23].

Learners’ preference for blocked study may also be moderated by metacognitive perceptions of learning. Learners often repeat study of items perceived as poorly learned, leading to blocked study, and defer study of those perceived as well learned, leading to interleaved study ([22,24], but see [25]). This behavior may result from a *discrepancy reduction* strategy [26], where knowledge that is furthest from some target is prioritized for study, or alternatively, where review of already-mastered items is deferred, as predicted by Metcalfe’s [27] region-of-proximal-learning framework. Although these studies employed mostly memorization paradigms (in which blocked study is verbatim repetition of the same item), they seem to indicate that learners’ preferences to block their study might be strategic and potentially beneficial. However, in studies in which learners cannot selectively block items perceived as poorly learned, these beneficial properties of blocked study are not available.

Successive examples may also be similar or dissimilar in their surface features. This distinction is independent of that between blocked and interleaved study of examples by concept; that is, successive examples may be either similar or dissimilar, regardless of whether they illustrate the same or different concepts. Should successive examples maximize similarity or highlight differences? In general, high similarity between examples facilitates identification of both similarities and differences through comparison [28], and so could increase the benefits of both blocked and interleaved study sequences. However, high variability between examples can promote generalization [29–33], and so could increase transfer of learning to novel cases following study. Thus, there are competing reasons to expect benefits from successive similar and varied examples. Currently, little is known as to how learners might choose to regulate similarity or variation between successive examples, nor how such learner regulation would affect learning outcomes.

### *In vivo* research of study practices

As we described in the previous sections, while existing research has provided considerable insight regarding the relative effectiveness of different study sequences in the laboratory, little is known about how learners behave in more ecologically valid contexts. Similarly, while previous studies have examined the effects of sequencing in classroom contexts [34–38], to the best of our knowledge the question has never been analyzed in a context where students have control over how to sequence their study. Study behavior that is maladaptive in the laboratory may be more effective in naturalistic situations [39,40]. For example, students may be more interested in the course content than in laboratory stimuli, more motivated to learn the material in order to get higher grades, more likely to monitor their own personal understanding of the material, and more distractible in natural studying environments. With these possibilities in mind, the primary goal of the present study is to investigate students’ choices regarding study sequencing of concepts and problem similarity in an ecologically valid context—a homework activity for a university course—and to test for associations of these choices with learning outcomes, measured via questions inserted into students’ mid-term exam.

In this experiment, students enrolled in an Introductory Psychology course were given practice calculating measures of central tendency using an online tutorial completed as homework following in-class instruction. The tutorial was composed of several problems on each of the measures of central tendency that students were studying in class. There were two groups of students. One group (Self-Regulated group) completed the tutorial by choosing which of three concepts—mean, median, or mode—to study for each successive problem, and whether or not to vary the problem’s content (background story and data). The other group of students (Yoked group) did not choose what to study but instead received study materials according to a sequence that another student in the Self-Regulated group had chosen.

For both groups, questions about measures of central tendency were included on a subsequent midterm exam which was part of their course evaluation and final grade. Exam scores were matched to records of tutorial usage to identify associations between study behavior and exam performance. Importantly, the tutorial and its content were part of the normal class activities and the outcome measures included in the exam contributed to the students’ overall grade in the class.

This kind of *in vivo* research in which manipulations of instructional practices are introduced into a real classroom context offer an exciting new frontier for valid and precise evaluation of educational practices [4]. *In vivo* research presents considerable ecological validity because it involves content belonging to the regular curriculum of a course and both intervention and assessment are highly integrated with the course, while maintaining the type of precise control of experimental manipulations required to extract valid conclusions [41].

**Fig 1:**
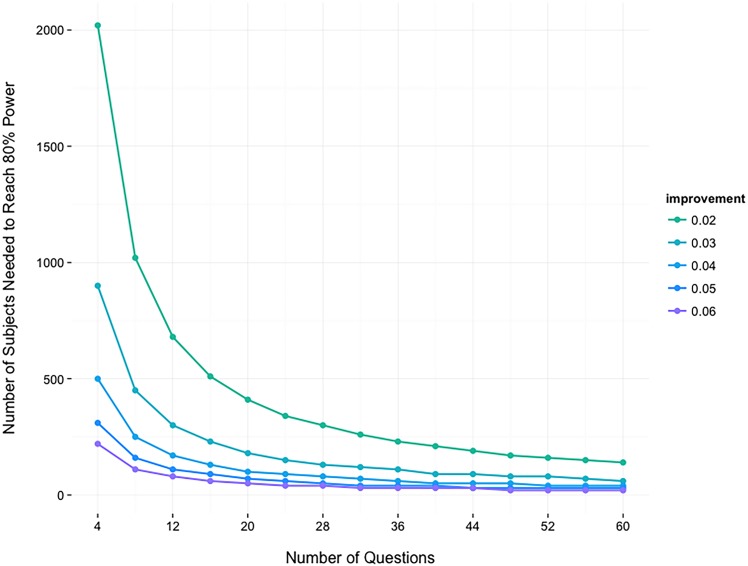
Number of participants needed for 80% power to detect posttest improvement, as a function of number of test questions and size of posttest improvement. The data reflect the results of simulated experiments in which participants completed a pretest and a posttest. We varied the number of questions appearing on each test (x-axis) and the mean improvement in accuracy from pretest to posttest (legend). For each combination of these variables, 10,000 simulated experiments were run for each of a range of sample sizes. The y-axis shows the smallest number of participants for which improvement from pretest to posttest was statistically significant (as determined by a paired *t*-test) in at least 80% of simulations. The results show the trade-off between the number of questions each individual participant responds to and the number of participants required to achieve adequate power.

One important characteristic of this type of research is that methodological decisions are tied to the environment in which the research will take place. As such, the materials used, the content used and the amount of test points available are constrained relative to lab studies. The materials must be coordinated with what instructors use in the classroom, the content needs to be part of the common curriculum, and students can only complete a few test questions due to time and instructional constraints. This last limitation, in particular, tends to reduce measurement precision for individual students, requiring larger samples to detect the same effects. As can be seen in the results of a simulation presented in Fig 1, to detect an average 4% improvement from pre to posttest using only four testing questions, a considerably higher number of subjects is needed than if a larger number of questions (in Fig 1, 60) were used in the tests, as is typical in laboratory studies. Because only four test questions could be used in the present study, we collected data from a large number of students in different classes and academic terms in an *in vivo* study that allowed only four exam questions testing trained material.

Data collection took place across two academic years: Spring 2013 (Time 1) and Fall 2014/Spring 2015 (Time 2). All students at Time 1 were included in the Self-Regulated group. At Time 2 participants were randomly assigned to either the Self-Regulated group or the Yoked group (see Methods below for details).

## Method

### Ethics Statement

All experimental protocols and consent materials were approved as an Exempt Study by the Indiana University Institutional Review Board. Instructors volunteered to include the study intervention as part of their Introductory Psychology curriculum, and all students of participating sections completed all the study procedures because they were part of the normal instructional activities of the class. A statement about this study was included in the syllabus of participating sections. Participant’s consent to have their data analyzed and included in this study was obtained from all students in compliance with the IRB of Indiana University. Parental consent for this study was waived by the IRB of Indiana University for students under the age of 18 enrolled in the class, because this activity was part of their curricular activities (and was specified as such on the syllabus), and a parent or guardian of any minor would have already consented to their participation in the course.

### Participants

A total of 2061 (Time 1: N = 678; Time 2: N = 1383) undergraduate students registered in one of the 18 sections of Introduction to Psychology at Indiana University participated in the study as part of their normal class activities and agreed to have their study data and grades included in the analyses for this study. The instructors of the participating section agreed to include the tutorial as an extra-credit homework activity and the posttest questions as part of their first evaluation point. Instructors were unware of the manipulation introduced (except for one section whose instructor was the fourth author). Some instructors taught more than one section in the same or across data collection times.

Students access the tutorial through a website, which randomly assigned them to either the Self-Regulated (N = 1386) or Yoked (N = 657) groups (see details below). Neither the student or the instructor had any *a priori* knowledge of the condition. The final sample of 2061 students includes 1208 students who were matched to another student (i.e., 604 complete Self-Regulated-Yoked dyads). The remaining students (N = 853) either did not have anybody paired with them or the student paired to them was not included in analyses due to not having consented or missing the exam.

Due to the nature of the research conducted and its inclusion as part of normal classroom activities, it was not possible to collect demographic information about the participating participants. However, to characterize the population we present demographic data for the population of students of Introductory Psychology at Indiana University Bloomington for the past 15 years in Table 1.

**Table 1:** Demographic information for the student population of Introductory Psychology at Indiana University Bloomington.

Variable	*M*	*SD*
High school GPA percentile score at admission (100 is best)	75.62	16.02
Age at beginning of first enrolled semester at IU	18.66	0.87
Cumulative undergraduate GPA	3.02	0.61
Percent reporting being first generation college students (binary)	0.10	0.29
Percent female (binary)	0.59	0.49
Ethnicity: Black	0.04	0.21
Ethnicity: Hispanic	0.02	0.14
Ethnicity: Asian	0.02	0.13
Indiana Resident	0.66	0.47
4-year graduation rate	0.54	0.50

Mean and standard deviations of several demographic measures for students enrolled in Introductory Psychology at Indiana University Bloomington over the past 15 years.

### Materials

Two sets of four multiple choice questions each were constructed, one of which served as pretest and the other as posttest. Each set consisted of two procedural questions requiring exact calculation of measures of central tendency and two conceptual questions requiring qualitative inferences about these measures (see Table 2). Two procedural and two conceptual questions were assigned to be used during the pretest while the remaining four were used in the posttest.

**Table 2:** Items used during pre- and posttest.

Procedural Questions	Conceptual Questions
Five cars were given safety ratings by consumer reports. Their ratings were: Spitfire = 3, Bentley = 7, Stanza = 8, Colt = 3, Lexus = 4. What are the mode, median and mean for this data set?	Imagine a difficult math test on which 13 students do very poorly, each getting a score of 1, 2 or 3 out of 100 possible points. However, the remaining 3 students get excellent scores: 96, 98, and 99. Will the mean be less than or more than the mode?
Three children in a family have ages of 7, 12, and 8. What are mean and median ages in this family?	There are 9 offensive players on a particular football team. On a particular game, the median number of yards gained by each player was 7 and no two players gained the same number of yards. If the worst and best performing offensive players are not considered, what will be the median of the remaining 7 players' gained yards?
Five pizzas were given quality scores by an expert taster. Their scores were: Pizza World = 8, Slices! = 3, Pisa Pizza = 2, Pizza a go-go = 4, Crusty's = 8. What are the mode, median and mean for this data set?	Imagine a vocabulary test in which 15 students do very well, getting scores of 98, 99, and 100 out of 100 possible points. However, the remaining 3 students get very poor scores: 5, 8, and 9. Will the mode be less than or more than the mean?
Three children in a family have shoe sizes of 5, 10, and 9. What are mean and median for the shoes sizes in this family?	There are 7 players on a particular basketball team. On a particular game, the median number of points scored by each player was 12 and no two players scored the same number of points. If the lowest and highest scoring players are not considered, what will be the median of the remaining 5 players' scores?

Two procedure and two conceptual questions were assigned to be used during the pretest while the remaining four were used in the posttest.

Thirty-two brief stories were designed for use as tutorial examples. Each story described a situation, such as “Several fishermen went fishing on the same day. Below you can find how many fish the different fishermen caught.” The situations varied in context and details. Each story also presented a data set generated pseudo-randomly online for each student according to the constraints of the situation and the students’ selections (see below for details).

Students completed the pretest and tutorial as an online homework activity, using an online platform created for this purpose. The exam was completed in a classroom at the time designated by the instructor and included a varying number of multiple-choice questions, four of which were included in all examinations for the purpose of this study.

### Procedure

**Fig 2:**
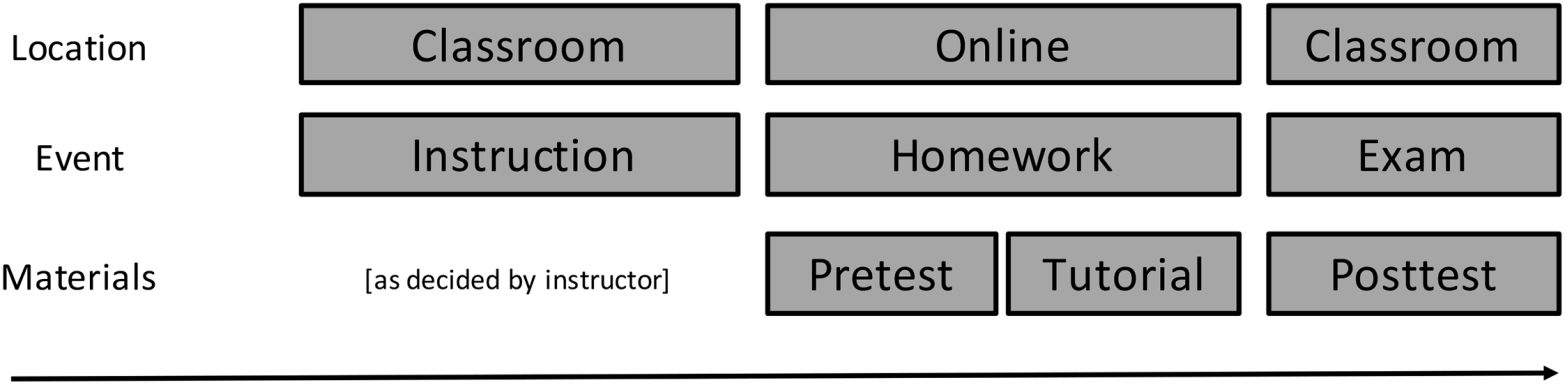
Schematic representation of the procedure for this study. Students were instructed on measures of central tendency by their instructor in the classroom during regular classroom activities. The online homework was then assigned. This homework started with 4 pretest questions about measures of central tendency without feedback and then proceeded with the tutorial. Four posttest question on measures of central tendency were included in each class’s regular mid-term exam.

An overview of the procedure is presented in Fig 2. During regular class sessions, students were introduced to measures of central tendency in the context of research in psychology and were assigned the pretest and tutorial as homework for class credit. They were also instructed that this homework assignment would be useful preparation for a future exam, which would include questions about measures of central tendency. The four posttest questions (see Table 2) were inserted into the standard midterm exam for the course. This exam was administered using paper and pencil during class sessions, at least two weeks after the homework was made available. All students were required to take this exam, regardless of whether they had done the homework.

Upon accessing the homework, students read a study information sheet informing them that the activity was part of study and requesting consent to have their data analyzed. Next, students completed the pretest questions one at a time, without feedback. The tutorial began immediately after completion of the pretest. Students first read a review describing how to calculate mean, median, and mode, followed by an explanation of the tutorial interface, followed by the tutorial problems.

**Fig 3:**
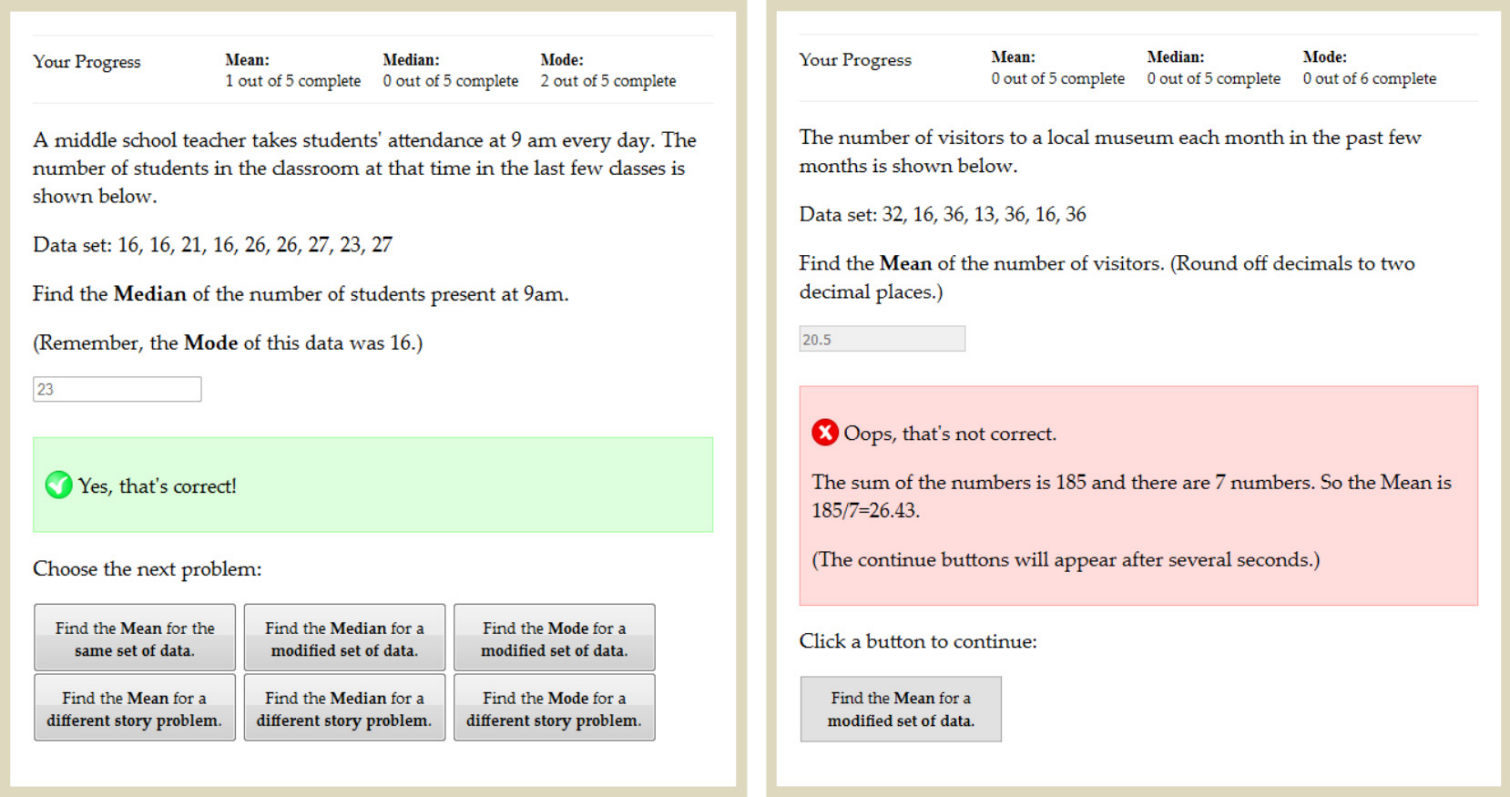
Tutorial interface for one of the trials during study. This example shows a problem and response feedback. The buttons at the bottom include all possible choices for the next problem. Left panel: Interface for the Self-Regulated group. Right panel: Interface for the Yoked group.

Each tutorial problem presented one story along with a dataset, and requested students to calculate the mean, median, or mode of the dataset (see Fig 3). For the first problem, the concept was always the mean, the story was selected randomly, and the dataset was generated quasi-randomly within the range specified for the story.

#### Self-regulated group

For students in the Self-Regulated group the concepts for subsequent problems (i.e., mean, median, or mode) were chosen by the students. Thus, students could choose to block study by studying each concept several times successively, to interleave by cycling through the three concepts repeatedly, or to adopt an intermediate approach.

Students could also determine the degree of similarity or variation between successive problems by choosing whether each subsequent problem would involve the same or a new story. If a new story was chosen, a new dataset was generated quasi-randomly. If the same story was chosen, the dataset for the next problem was either identical to the current dataset if the concept for the next problem had not been probed yet with that dataset, or a modified version of the current dataset otherwise.

As shown in Fig 3, the current story and dataset for each problem were displayed near the top of the screen, followed by an instruction to calculate mean, median, or mode. Once a response was submitted, feedback was displayed indicating whether the response was correct, followed by two rows of buttons, which were used by students to choose what type of problem would appear next (see left panel of Fig 3). The order and positions in which the buttons appeared was constant across trials and across participants.

Problems involving the same or modified versions of the datasets used in the preceding problems also included prompts intended to facilitate comparison with the preceding problems. First, reminders of the answers to the preceding problems were displayed just above the response area. For example, the problem shown in the left panel of Fig 3 requests the median, but includes a reminder of the answer to the previous problem, i.e. the mode. Second, problems involving modified datasets included descriptions of how the datasets were modified, with additions and deletions marked in the data.

The number of problems already completed for each concept was displayed at the top of the screen. Students were informed that they had to complete at least 5 problems for each concept (regardless of correctness of responses). After this criterion was met, an option to end the tutorial became available. However, students could continue the tutorial as long as they wished. Students were encouraged to use a calculator, and a link to an online calculator was provided.

#### Yoked group

For students in the Yoked Group the procedure was similar in all aspects except that during the tutorial students could not choose the concept or the story of the next problem. Following a response to a problem, students saw only a button to advance to the next problem (see right panel of Fig 3). In order to equate the groups on as many variables as possible we used a Yoked design. Each student in the Yoked Group was yoked to a student in the Self-Regulated Group who had previously completed the tutorial, and received the same sequence of study examples as that chosen by the student to whom they were yoked. Thus, students in the Yoked Group completed the same number of each type of problem in the same sequence as those in the Self-Regulated Group, and differed only in that they did not choose the problems themselves. Students in the two groups were matched based on their pretest scores to equate for initial levels of mastery.

## Results

Descriptive statistics of several study behavior measures for both groups are shown in Table 3. No differences were found between the two groups for any of the descriptive measures considered. Overall, average pretest performance was 72.4%. Posttest scores were overall higher (*M* = 84.3%), *t* (2060) = 21.57, *p* < .0001. On average students completed a total of 17 problems during the tutorial, with high accuracy (*M* = 90%).

**Table 3:** Summary statistics for study behavior for the Self-Regulated and Yoked groups and *t*-tests statistics comparing differences between the two groups.

Measure	Self-Regulated Group Mean (SD)	Yoked Group Mean (SD)	*t*-stats	*p*-value
Pretest Score	72% (24)	73% (24)	1.42	.15
Posttest Score	84% (20)	84% (20)	0.53	.60
Mean Exam Score (z-score)	51 (9.5)	51 (9.6)	0.70	.49
# practice trials	17 (2)	17 (1)	0.05	.96
Tutorial accuracy	90% (12)	91% (10)	1.63	.10
Concept Repetition Rate	64% (29)	65% (29)	0.80	.42
Story Repetition Rate	75% (26)	76% (26)	0.32	.75
Concept avg. block length	4 (2)	4 (2)	0.98	.33
Study-Test lag (days)	10 (6)	9 (7)	0.55	.58

Mean values and corresponding standard deviations (in parenthesis) of different measures for both groups in the study. Student’s *t*-test statistics and corresponding *p* values are presented in the last two columns. All *t*-tests were between-subject analyses with 2059 degrees of freedom.

How did students in the Self-Regulated group organize their study? Students overwhelmingly preferred to repeat content in successive problems. We use concept and story repetition rates to illustrate this; other measures yield similar results. On average, students in the Self-Regulated group chose to repeat the same concept on 64% of the problems (compared to a random repetition rate of 33%). Similarly, on average, students chose to repeat the same story on 75% of of the problems. The rates of concept and story repetition for the Yoked group were nearly identical to those of the Self-Regulated group, as expected due to the yoked design.

There are several reasons why students might prefer to repeat similar material on successive study trials. One possibility is that these choices are strategic and stem from students’ desire to practice what they have not yet mastered. If so, students’ decisions to repeat similar material or not should be sensitive to their perceived mastery of the material. To test this possibility, concept and story repetition rates in the Self-Regulated group were calculated separately for transitions following correct and incorrect responses for each participant, excluding those who gave no incorrect or no correct responses before any transition. In the Self-Regulated group, students were more likely to repeat the same concept after a problem they solved incorrectly (*M* = 72%, *SD* = 36) than following a problem they solved correctly (*M* = 66%, *SD* = 29), *t* (930) = 4.64, *p* < .0001. Similarly, students were more likely to repeat the same story following an incorrect response (*M* = 78%, *SD* = 35) than a correct one (*M* = 75%, *SD* = 28), *t* (930) = 3.71, *p* = .0002. Students in the Yoked group did not have the opportunity to choose the next problem to study, so correctness did not affect concept or story repetition rates in this group.

How did study sequence relate to learning, as measured by subsequent exam performance? Notwithstanding the strategic nature of students’ choices, it is possible that the particular sequences chosen were not optimal for study or did not have a long-term effect on students’ later exam performance. To look at this possibility, the posttest accuracy data were analyzed with multiple linear regression using pretest score, study group, concept repetition rate, story repetition rate, number of tutorial trials, and tutorial accuracy as predictors, as well as interactions factors for the interaction between concept repetition rate, story repetition rate and study group. To correct for skewedness in the distribution of responses, pre- and posttest scores were submitted to an empirical Logit transformation for this and subsequent analyses. The other predictors were centered (by subtracting the average of the population to each score) before being entered into the model.

The model was significant, accounting for 12.2% of the variance in posttest score, *F* (10, 2050) = 29.65, *p* < .0001. The coefficients and significance of the various predictors are displayed in Table 4.

**Table 4:** Results of Regression Analysis of Posttest Accuracy (*N* = 2061).

Predictor	B	*SE* B	*β*	*p*-value
Constant	0.166	0.004		
Pretest Score	0.284	0.018	0.337	< .0001*
Tutorial Accuracy	0.005	0.003	0.039	.069
Number of Tutorial Problems	- 0.005	0.003	-0.040	.068
Concept Repetition Rate	0.001	0.003	0.008	.724
Story Repetition Rate	0.001	0.003	0.013	.608
Study Group	0.003	0.003	0.012	.249
Concept Rep. Rate *X* Story Rep. Rate	-0.001	0.004	-0.001	.754
Concept Repetition Rate *X* Study Group	0.006	0.003	0.046	.039*
Story Rep. Rate *X* Study Group	0.001	0.003	0.008	.748
Concept Rep. Rate *X* Story Rep. Rate *X* Study Group	-0.003	0.004	-0.024	.432

This table presents the estimates (B) and corresponding standard errors (*SE* B) as well as standardized coefficients (*β*) and corresponding significance value for each predictor and interaction entered in the multiple regression model of posttest accuracy. Asterisks indicate p < .05.

Not surprisingly, participants who scored well on the pretest also scored well on the posttest, as indicated by a significant effect of pretest score, uniquely accounting for 12% of the variance in posttest score, *β* = 0.337, *t* (2050) = 15.76, *p* < .0001, partial *r*^2^ = 0.120. Moreover, a significant interaction between concept repetition rate and study group was also found, uniquely accounting for 0.17% of the variance in posttest score, *β* = 0.046, *t* (2050) = 2.06, *p* = .039, partial *r*^2^ = 0.0017. The effects of the other predictors were not significant, all *p*s>.07. A similar pattern of results was found when analyzing separately the results for conceptual and procedural exam questions and when taking into account only students belonging to yoked pairs in which both students participated during the same semester and provided complete data.

**Fig 4:**
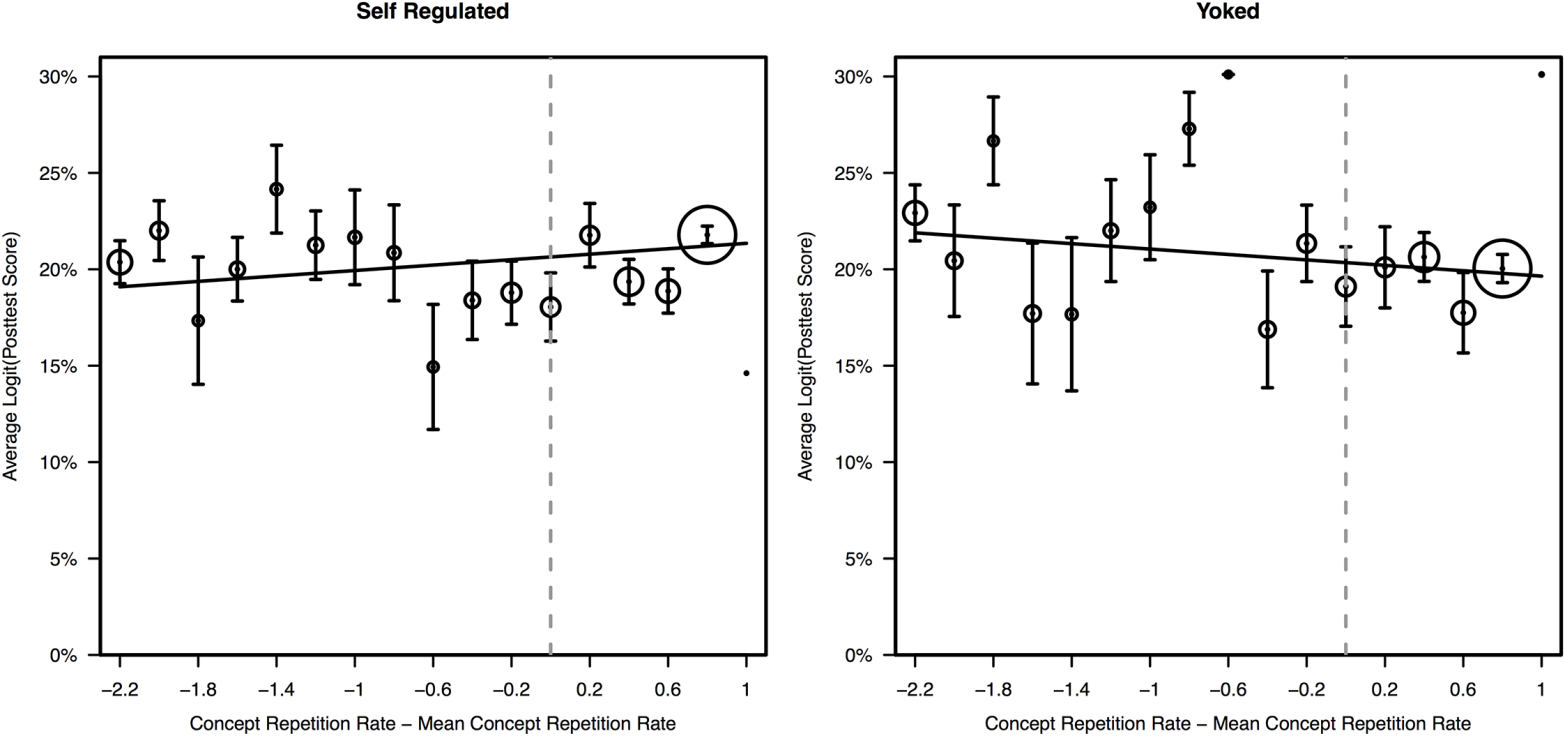
Average Posttest score by concept repetition rate for the Self-Regulated (left panel) and Yoked (right panel) groups. Students were divided into bins by their adjusted rate of repetition and average posttest scores (Logit transformed) within each bin were plotted. Concept repetition rate was adjusted by subtracting the average rate of repetition for the entire group from the rate of repetition for the bin—a value of 0 in the x-axis indicates mean concept repetition rate (represented by the vertical dashed line) and increasing values indicate increasing difference from average. The values in the y-axis represent Logit transformed posttest scores. Each point in the graph lies at the center of a 20%-wide interval of concept repetition rates, and represents the average posttest score among students whose concept repetition rates fell in that interval. The number of students in each bin is represented by the area of the circles surrounding the data points. The regression lines represent best fitting lines of the regression analyses assuming average values for all predictors other than concept repetition rate.

To investigate the interaction between study group and concept repetition rate further, we plotted average posttest score (Logit transformed) by centered concept repetition rate for each study group separately (Fig 4). As can be seen from Fig 4, when students decide how to organize their study, higher rates of repetition were associated with higher posttest scores. However, this was not the case when students did not choose how to organize their study, even though this group experienced identical rates of concept repetition as their Self-Regulated counterparts. Separate multiple regression analyses for each group confirm this interpretation: Repetition rate had a significant positive effect on posttest scores for the Self-Regulated group, *β* = 0.060, *t* (1308) = 2.27, *p* = .023, partial *r*^2^ = 0.003, but not for the Yoked group, *β* = -0.057, *t* (669) = -1.45 *p* = .146, partial *r*^2^ = 0.003.

## Discussion

The way study materials are sequenced influences learning [8,9,14,20,34,36,37,42]. The present work adds to previous findings by (1) demonstrating an influence of study sequence on learning outcomes in a classroom context with class relevant materials and testing situations, and (2) demonstrating that similar sequences of study might have different impacts depending on whether they are chosen by the students or pre-determined for them.

When students choose how to organize their study they show a clear tendency to block their study by concept. This result replicates the findings of Tauber et al. [21], and extends them to an ecologically valid context: Students studying in preparation for a test. As mentioned in the Introduction, although this preference could reflect a belief that blocked study leads to better learning [3,23], it could also reflect a habitual bias towards blocked study [22] or an avoidance of extra work [23].

Students also predominantly chose to study similar successive examples, even across different concepts. Story repetition may have been preferred because it led to more fluent processing during study and thus increased self-perceptions of learning. Alternatively, participants may have wished to compare successive examples, and found this easier to do when successive examples involved similar content [28], both between and within categories. However, we found no evidence that story repetition resulted in improved learning, as both these possibilities would suggest, or any interacted with concept repetition.

Furthermore, concept and story repetition rates were higher following incorrect than correct responses for students who choose how to organize their study. This result may reflect metacognitive influences on study regulation, that is, students might have perceived the current category as less well-learned following incorrect than correct responses, and thus chosen to repeat both concepts and stories more often in the former case. Thus, our results are in line with previous findings that learners tend to defer study of well learned items, and to immediately repeat study of poorly learned ones [22,24,43]. Students may have preferentially repeated concepts perceived to be far from a target level of mastery [26] or alternatively, avoided repetition when it was expected to yield little incremental benefit [27].

Regardless of why students choose to frequently repeat concepts on successive problems, one important question is whether this decision had an impact on performance in subsequent outcome measures. When sequence of study was assigned to students, we found no relation between concept repetition rates during the tutorial and posttest scores. These findings are consistent with the possibility that blocked study can be beneficial for learning when students themselves choose it.

These analyses are correlational and, therefore, do not establish a causal connection between high concept repetition and learning outcomes. However, demonstrating such a causal connection by an experimental manipulation of study sequence would be impossible if the connection is contingent on students being in control of their own study sequence. In other words, we cannot assign a study sequence when students are given the ability to choose a study sequence. Moreover, in the Self-Regulated group, concept repetition rate was not correlated with pretest score, *r* = -.041, *p* = .129, and the association of concept repetition with posttest score was significant even after controlling for pretest score. These facts argue against an explanation of the effect of category repetition rate in terms of differences in pre-existing ability or diligence during the tutorial.

It is important to note the small magnitude of this interaction effect between study group and concept repetition rate on posttest performance (*β* = 0.046, partial *r*^2^ = 0.002, *p* = .034), which recommends caution in interpreting the results. Several factors are likely to have contributed to the small effect size found. As mentioned in the introduction, the *in vivo* nature of this study limited the resolution of the measurement used with only four posttest questions and naturally includes a large degree of variability. Moreover, because most students showed high levels of knowledge about the target content of this study at pretest, the range of possible improvement was compressed.

Despite the small effect size, the results found do suggest that students who choose to block their study by concept can show greater improvements. These results contrast with previous studies that have found superior learning from interleaved study [7,8,10,14,37,38,44]. However, previous studies did not consider the effect of self-regulation on the effectiveness of study sequence. Why might self-regulated learning conditions increase the relative effectiveness of blocked study?

The reasons may have to do with changes in the attentional and cognitive processing requirements of study under self-regulated conditions. First, a commonly mentioned drawback of blocked study is its repetitive nature, which might result in attention attenuation [10]. Because interleaved study requires learners to provide different answers or applying different procedures in consecutive problems, it inherently involves greater attentional involvement [38]. Similarly, the requirement to choose the next problem during self-regulated learning might involve a more thorough analysis of the learning materials and greater engagement [45–47], and require greater attention and effort during encoding [e.g., 48]. These factors could alleviate an important weakness of blocked study and thus increase its relative effectiveness.

Second, interleaved study requires students to keep active their knowledge of several procedures or concepts simultaneously, which might result in greater cognitive load [49–52] and change their perceptions of mastery, negatively impacting their study decisions [53]. These potential drawbacks of interleaved study could have a more serious effect when compounded by the increased cognitive demands of self-regulated study described above. By contrast, in blocked study, the task is not as demanding on cognitive resources because students can focus on one concept at a time. Thus, self-regulated conditions could exacerbate a potential weakness of interleaved study. In sum, self-regulated study could mitigate problems associated with blocked study, aggravate problems associated with interleaved study, or both.

## Conclusions

In recent years, there has been an increasing interest on how the sequence of study can affect learning [7–9,13,14,37,54] in no small part because students are increasingly in charge of organizing their own study, and may fail to adopt the optimal sequence of study [3] with potential negative consequences. In this context, we believe the current research raises two important points.

First, it is important to consider the possibility that apparently inefficient study decisions might be beneficial if the student is the one making them. Despite the large literature showing an advantage for interleaved study in inductive category learning, the present work points to possible advantages for blocked study. Importantly, the benefits of blocked study were only found when students actively chose it themselves rather than having it imposed on them. We believe more work is needed to investigate how the best sequence may depend on the study situation, and the importance of self-regulation in the context of studying decisions.

Secondly, from a methodological point of view, this experiment can serve as a model for carefully controlled research in naturalistic contexts and pedagogically relevant issues. The effect of self-regulation described here, albeit small, was found in the context of students’ real study experience, very different from the common laboratory study. Although this methodology might present lower measurement resolution (only four test questions) and increased measurement noise resulting in decreased power compared to the usual laboratory study, it has considerable ecological validity. Moreover, in contrast to many classroom studies, online distribution of the tutorial allowed considerable control over the intervention and precise recording of the students’ behavior for inclusion in subsequent analyses. We believe that similar *in vivo* yet individually-controlled studies of learning in educational contexts [41,55] represent a major potential growth area for cognitive science.

## References

[1] Gureckis TM, Markant DB. Self-Directed Learning: A Cognitive and Computational Perspective. Perspect Psychol Sci. 2012;7: 464–481. 10.1177/1745691612454304 26168504

[2] Tullis JG, Benjamin AS. On the effectiveness of self-paced learning. J Mem Lang. Elsevier Inc.; 2011;64: 109–118. 10.1016/j.jml.2010.11.002 PMC3079256 21516194

[3] Bjork RA, Dunlosky J, Kornell N. Self-regulated learning: beliefs, techniques, and illusions. Annu Rev Psychol. 2013;64: 417–444. 10.1146/annurev-psych-113011-143823 23020639

[4] Koedinger KR, Booth JL, Klahr D. Instructional Complexity and the Science to Constrain It. Science (80-). 2013;342: 935–937. 10.1126/science.1238056 24264979

[5] Elio R, Anderson JR. The effects of information order and learning mode on schema abstraction. Mem Cognit. 1984;12: 20–30. 6708807 10.3758/bf03196994

[6] Medin DL, Bettger JG. Presentation order and recognition of categorically related examples. Psychon Bull Rev. 1994;1: 250–254. 10.3758/BF03200776 24203473

[7] Birnbaum MS, Kornell N, Bjork EL, Bjork RA. Why interleaving enhances inductive learning: The roles of discrimination and retrieval. Mem Cognit. 2013;41: 392–402. 10.3758/s13421-012-0272-7 23138567

[8] Kornell N, Bjork RA. Learning concepts and categories: is spacing the “enemy of induction”? Psychol Sci; 2008;19: 585–592. 10.1111/j.1467-9280.2008.02127.x 18578849

[9] Rohrer D, Taylor K. The shuffling of mathematics problems improves learning. Instr Sci. 2007;35: 481–498.

[10] Wahlheim CN, Dunlosky J, Jacoby LL. Spacing enhances the learning of natural concepts: an investigation of mechanisms, metacognition, and aging. Mem Cognit. 2011;39: 750–763. 10.3758/s13421-010-0063-y 21264639 PMC3085105

[11] Carvalho PF, Goldstone RL. What you learn is more than what you see: what can sequencing effects tell us about inductive category learning? Front Psychol. 2015;6: 1–12. 25983699 10.3389/fpsyg.2015.00505 PMC4415402

[12] Bjork RA. Memory and metamemory considerations in the training of human beings In: Metcalfe J, Shimamura AP, editors. Metacognition: Knowing about knowing. Cambridge, MA: MIT Press; 1994 pp. 185–205.

[13] Carvalho PF, Goldstone RL. The benefits of interleaved and blocked study: Different tasks benefit from different schedules of study. Psychon Bull Rev. 2015;22: 281–288. 10.3758/s13423-014-0676-4 24984923

[14] Carvalho PF, Goldstone RL. Putting category learning in order: Category structure and temporal arrangement affect the benefit of interleaved over blocked study. Mem Cognit. 2014;42: 481–495. 10.3758/s13421-013-0371-0 24092426

[15] Goldstone RL. Isolated and interrelated concepts. Mem Cognit. 1996;24: 608–628. 8870531 10.3758/bf03201087

[16] Kang SHK, Pashler H. Learning Painting Styles: Spacing is Advantageous when it Promotes Discriminative Contrast. Appl Cogn Psychol. 2012;26: 97–103.

[17] Carvalho PF, Goldstone RL. Effects of interleaved and blocked study on delayed test of category learning generalization. Front Psychol. 2014;5: 1–11. 25202296 10.3389/fpsyg.2014.00936 PMC4141442

[18] Carpenter SK, Mueller FE. The effects of interleaving versus blocking on foreign language pronunciation learning. Mem Cognit. 2013;41: 671–682. 10.3758/s13421-012-0291-4 23322358

[19] Kurtz KH, Hovland CI. Concept learning with differing sequences of instances. J Exp Psychol. 1956;51: 239–243. 13306871 10.1037/h0040295

[20] Zulkiply N, Burt JS. The exemplar interleaving effect in inductive learning: Moderation by the difficulty of category discriminations. Mem Cognit. 2013;41: 16–27. 10.3758/s13421-012-0238-9 22886736

[21] Tauber SK, Dunlosky J, Rawson KA, Wahlheim CN, Jacoby LL. Self-regulated learning of a natural category: Do people interleave or block exemplars during study? Psychon Bull Rev. 2013;20: 3560363. 10.3758/s13423-012-0319-6 23055143

[22] Pyc MA, Dunlosky J. Toward an understanding of students’ allocation of study time: why do they decide to mass or space their practice? Mem Cognit. 2010;38: 431–40. 10.3758/MC.38.4.431 20516223

[23] Son LK, Simon D a. Distributed Learning: Data, Metacognition, and Educational Implications. Educ Psychol Rev. 2012;24: 379–399.

[24] Son LK. Spacing one’s study: Evidence for a metacognitive control strategy. J Exp Psychol Learn Mem Cogn. 2004;30: 601–4. 10.1037/0278-7393.30.3.601 15099128

[25] Benjamin AS, Bird RD. Metacognitive control of the spacing of study repetitions. J Mem Lang. 2006;55: 126–137. 10.1016/j.jml.2006.02.003

[26] Dunlosky J, Hertzog C. Training programs to improve learning in later adulthood: Helping older adults educate themselves In: Hacker DJ, Dunlosky J, Graesser AC, editors. Metacognition in educational theory and practice The educational psychology series. Mahwah, NJ: Lawrence Erlbaum Associates; 1998 pp. 249–275.

[27] Metcalfe J. Metacognitive judgments and control of study. Curr Dir Psychol Sci. 2009;18: 159–163. 10.1111/j.1467-8721.2009.01628.x 19750138 PMC2742428

[28] Gentner D, Markman AB. Structural Alignment in Comparison—No Difference Without Similarity. Psychol Sci. 1994;5: 152–158. 10.1111/j.1467-9280.1994.tb00652.x

[29] Posner MI, Keele SW. On the Genesis of Abstract Ideas. J Exp Psychol. 1968;77: 353–363. 5665566 10.1037/h0025953

[30] Perry LK, Samuelson LK, Malloy LM, Schiffer RN. Learn Locally, Think Globally: Exemplar Variability Supports Higher-Order Generalization and Word Learning. Psychol Sci. 2010;21: 1894–1902. 10.1177/0956797610389189 21106892 PMC3144952

[31] Gómez R. Variability and detection of invariant structure. Psychol Sci. 2002;13: 431–437. 12219809 10.1111/1467-9280.00476

[32] Braithwaite DW, Goldstone RL. Inducing mathematical concepts from specific examples: The role of schema-level variation. In: Miyake N, Peebles D, Cooper RP, editors. Proceedings of the 34th Annual Conference of the Cognitive Science Society. Austin, TX: Cognitive Science Society; 2012. pp. 138–143.

[33] Braithwaite DW, Goldstone RL. Effects of Variation and Prior Knowledge on Abstract Concept Learning. Cogn Instr. 2015;33: 226–256. 10.1080/07370008.2015.1067215

[34] Horn S, Hernick M. Improving student understanding of lipids concepts in a biochemistry course using test-enhanced learning. Chem Educ Res Pract. Royal Society of Chemistry; 2015; 10.1039/C5RP00133A

[35] Ostrow K, Heffernan N, Heffernan C, Peterson Z. Artificial Intelligence in Education. In: Conati C, Heffernan N, Mitrovic A, Verdejo MF, editors. 17th International Conference on Artificial Intelligence in Education. 2015. pp. 338–347. 10.1007/978-3-319-19773-9

[36] Rau MA, Aleven V, Rummel N. Interleaved practice in multi-dimensional learning tasks: Which dimension should we interleave? Learn Instr. 2013;23: 98–114.

[37] Rohrer D, Dedrick RF, Stershic S. Interleaved Practice Improves Mathematics Learning. 2015;107: 900–908.

[38] Rohrer D, Dedrick RF, Burgess K. The benefit of interleaved mathematics practice is not limited to superficially similar kinds of problems. Psychon Bull Rev. 2014;21: 1323–1330. 10.3758/s13423-014-0588-3 24578089

[39] Bronfenbrenner U. The Experimental Ecology of Education. Educ Res. 1976;5: 5–15.

[40] Newman D, Cole M. Can Scientific Laboratory be Research From of Any Use to the Teachers? 2010;43: 260–267.

[41] Koedinger KR, Aleven V, Roll I, Baker R. In vivo experiments on whether supporting metacognition in intelligent tutoring systems yields robust learning In: Hacker DJ, Dunlosky J, Graesser AC, editors. Handbook of Metacognition in Education. New York, NY: Routledge; 2009 pp. 647–51. 10.1002/lsm.20954

[42] Rawson KA, Thomas RC, Jacoby LL. The Power of Examples: Illustrative Examples Enhance Conceptual Learning of Declarative Concepts. Educ Psychol Rev. 2015;27: 483–504. 10.1007/s10648-014-9273-3

[43] Son LK. Metacognitive control and the spacing effect. J Exp Psychol Learn Mem Cogn. 2010;36: 255–62. 10.1037/a0017892 20053063

[44] Taylor K, Rohrer D. The effects of interleaved practice. Appl Cogn Psychol. 2010;24: 837–848.

[45] Kimball DR, Metcalfe J. Delaying judgments of learning affects memory, not metamemory. Mem Cognit. 2003;31: 918–929. 10.3758/BF03196445 14651299

[46] Leotti LA, Ochsner KN. Born to Choose: The Origins and Value of the Need for Control Lauren. Trends Cogn Sci. 2010;14: 457–463. 20817592 10.1016/j.tics.2010.08.001 PMC2944661

[47] Kaplan R, Doeller CF, Barnes GR, Litvak V, Düzel E, Bandettini PA, et al Movement-Related Theta Rhythm in Humans: Coordinating Self-Directed Hippocampal Learning. PLoS Biol. 2012;10: 1–13. 10.1371/journal.pbio.1001267 PMC3289589 22389627

[48] Voss JL, Gonsalves BD, Federmeier KD, Tranel D, Neal J. Hippocampal brain-network coordination during volitional exploratory behavior enhances learning. Nat Neurosci. 2011;14: 115–120. 21102449 10.1038/nn.2693 PMC3057495

[49] de Croock MB., van Merriënboer JJG, Paas FGW. High versus low contextual interference in simulation-based training of troubleshooting skills: effects on transfer performance and invested mental effort. Comput Human Behav. 1998;14: 249–267. 10.1016/S0747-5632(98)00005-3

[50] van Merriënboer JJG, Sweller J. Cognitive Load Theory and Complex Learning: Recent Developments and Future Directions. Educ Psychol Rev. 2005;17: 147–177. 10.1007/s10648-005-3951-0

[51] Hall KG, Domingues DA, Cavazos R. Contextual interference effects with skilled baseball players. Percept Mot Skills. Ammons Scientific; 1994;78: 835–841. 10.1177/003151259407800331 8084699

[52] van Merriënboer JJG, de Croock MB., Jelsma O. The transfer paradox: Effects of contextual interference on retention and transfer performance of a complex cognitive skill. Percept Mot Skills. 1997;84: 784–786. 10.2466/pms.1997.84.3.784

[53] Josephs RA, Silvera DH, Giesler RB. The Learning Curve as a Metacognitive Tool. J Exp Psychol Learn Mem Cogn. 1996;22: 510–524.

[54] Li N, Cohen WW, Koedinger KR. Problem order implications for learning. Int J Artif Intell Educ. 2013;23: 71–93. 10.1007/s40593-013-0005-5

[55] Day SB, Motz BA, Goldstone RL. The cognitive costs of context: The effects of concreteness and immersiveness in instructional examples. Front Psychol. 2015;6 10.3389/fpsyg.2015.01876 PMC4665226 26648905

